# Toxicity after radiochemotherapy for glioblastoma using temozolomide - a retrospective evaluation

**DOI:** 10.1186/1748-717X-6-141

**Published:** 2011-10-21

**Authors:** Marcus Niewald, Christian Berdel, Jochen Fleckenstein, Norbert Licht, Ralf Ketter, Christian Rübe

**Affiliations:** 1 Department of Radiotherapy and Radiation Oncology, Saarland University Hospital, Kirrberger Straße, D-66421 Homburg, Germany; 2 Department of Neurosurgery, Saarland University Hospital, Kirrberger Straße, D-66421 Homburg, Germany

**Keywords:** Glioblastoma, Radiotherapy, Chemotherapy, Toxicity, Bone marrow

## Abstract

**Purpose:**

Retrospective evaluation of toxicity and results after radiochemotherapy for glioblastoma.

**Methods:**

46 patients with histopathologically proven glioblastoma received simultaneous radiochemotherapy (RCT). The mean age at the beginning of therapy was 59 years, the mean Karnofsky performance index 80%. 44 patients had been operated on before radiotherapy, two had not. A total dose of 60 Gy was applied in daily single fractions of 2.0 Gy within six weeks, 75 mg/m^2^/day Temozolomide were given orally during the whole radiotherapy period.

**Results:**

A local progression could be diagnosed in 34/46 patients (70%). The median survival time amounted to 13.6 months resulting in one-year and two-year survival probabilities of 48% and 8%, respectively.

Radiotherapy could be applied completely in 89% of the patients. Chemotherapy could be completed according to schedule only in 56.5%, the main reason being blood toxicity (50% of the interruptions). Most of those patients suffered from leucopenia and/or thrombopenia grade III and IV CTC (Common toxicity criteria). Further reasons were an unfavourable general health status or a rise of liver enzymes.

The mean duration of thrombopenia and leucopenia amounted to 64 and 20 days. In two patients, blood cell counts remained abnormal until death. In two patients we noticed a rise of liver enzymes. In one of these in the healing phase of hepatitis a rise of ASAT and ALAT CTC grade IV was diagnosed. These values normalized after termination of temozolomide medication. One patient died of pneumonia during therapy.

**Conclusion:**

Our survival data were well within the range taken from the literature. However, we noticed a considerable frequency and intensity of side effects to bone marrow and liver. These lead to the recommendations that regular examinations of blood cell count and liver enzymes should be performed during therapy and temozolomide should not be applied or application should be terminated according to the criteria given by the manufacturer.

## Background

Since the randomized trial published by Stupp et al. [1] in 2005 simultaneous radiochemotherapy applying a total dose of 60 Gy in 30 fractions of 2 Gy each within six weeks and temozolomide in a dosage of 75 mg/m^2^/day is regarded to be the gold standard in the treatment of glioblastoma. However, Stupp et al. [1] observed considerable side-effects such as fatigue, bone marrow suppression, opportunistic infections, cerebral hemorrhage, or liver irritation [1]. They reported a rate of neutropenia and thrombopenia grade III and IV CTC 2.0 (Common toxicity criteria) of 7%. Other author groups have published case reports with prolonged pancytopenia and isolated thrombopenia [2-4]. Additionally, single cases of liver damage by temozolomide have been reported [5].

During the review of our data we had the impression that a noticeably higher proportion of our patients suffered from prolonged leucopenia and thrombopenia than mentioned in the literature. This led us to a more detailed analysis of our data concerning toxicity of simultaneous radiochemotherapy for glioblastoma.

## Methods

From July 2000 to December 2009 a total of 46 patients underwent simultaneous radiochemotherapy for glioblastoma. Follow-up data were taken into account until December 2009. The patient characteristics are summarized in Table 1. The mean time interval between histology and start of radiotherapy amounted to 27 days.

**Table 1:** Patient characteristics

Item	Mean value/No. of patients	%
Gender		

Male	26	57

Female	20	43

		

Mean age	58.6 [33.2-73] years	

		

Mean Karnofsky performance index	81% [60-100%]	

		

Localization		

frontal	10	22

temporal	26	56

parietal	5	11

occipital	3	7

basal ganglia	1	2

multifocal	1	2

		

Mean diameter of tumour	4.3 [1.5-8.0] cm	

		

Size of edema		

none	5	11

< 1 cm	6	13

> 1 cm	15	33

half hemisphere	18	39

total hemisphere	2	4

		

Pre-treatment		

complete resection	27	59

subtotal resection	5	11

partial resection	12	26

biopsy	2	4

		

Corticoid intake (beginning of therapy)		

None	0	0

< 5 mg/day	1	2

< 10 mg/day	14	30

< 20 mg/day	21	46

< 30 mg/day	2	4

> 30 mg/day	8	18

The patient's head was fixed by an individual mask, CT- and MRI-scans (often MPRage and T2 series) were performed and fused in the 3D-planning system. The planning target volume (PTV) was delineated afterwards, it consisted of the tumour (bed) with a safety margin of 1.5 - 2 cm in all directions respecting anatomical barriers which prevent tumour extension like the scull or the falx cerebri. Furthermore, critical structures like brainstem, optic chiasm, optic nerves, lenses and the inner ear were contoured, the tolerance doses of those organs were taken into account (dose-volume histogram). A 3D conformal multiple-field technique applying 6 MV photons of a linear accelerator was used. A total dose of 60 Gy was applied in single fractions of 2.0 Gy once a day and five times a week using 6 MV photons of a linear accelerator.

Chemotherapy was applied simultaneously in all patients. They received 75 mg/m^2 ^Temozolomide daily during the whole duration of radiotherapy. In order to treat nausea and vomiting early, the patients were treated in an inpatient setting for the first few days of therapy. Laboratory tests (blood cell count, liver enzymes) were performed once or twice a week, chemotherapy was interrupted or even terminated in patients showing white blood cell counts below 3000/μl and/or platelet counts below 100.000/μl. Trimethoprim/Sulfamethoxazole were regularly given as a prophylaxis against pneumonia caused by Pneumozystis carinii infection.

The first follow-up examination was performed 6-8 weeks after the end of radiotherapy, after that twice a year, consisting of clinical examination and MRT/CT scans. Side-effects were graded according to the CTC 3.0 system [6]. Further examinations were performed in the Department for Neurosurgery. The diagnosis of a progression was performed according to the RECIST (response evaluation criteria in solid tumours) criteria. In order to improve the completeness of data concerning survival, local tumour result, and side effects, structured questionnaires were sent to the patients' local doctors and the local authorities.

All data were entered into a medical databank (MEDLOG, Fa.Parox, Münster, Germany) and evaluated statistically. Survival curves were computed using the Kaplan-Meier estimate, we tried to find out significantly independent prognostic factors using the Cox regression hazard model.

All patients had given their written informed consent before radiochemotherapy. An approval by the local ethics committee was not necessary due to the retrospective nature of this evaluation. The research carried out here is in compliance with the declaration of Helsinki.

## Results

**Figure 1:**
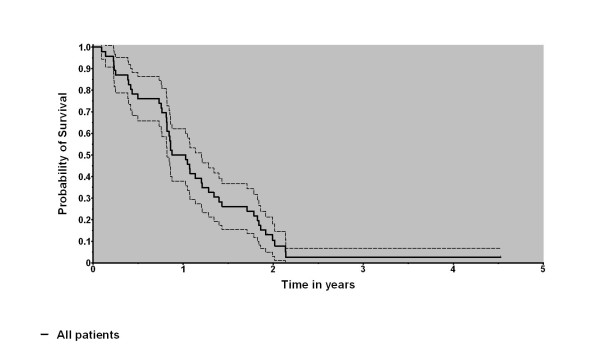
**Overall survival (Kaplan-Meier estimate, survival curve solid, borders of 95% interval dashed)**.

Median overall survival amounted to 13.6 months, with one/two year survival rates of 48 and 8%, respectively. The data are depicted in Figure 1. Local tumour progression was seen in 32 patients (82%) resulting in a 6/12 months local control probability of 59.4 and 30%, respectively. In multivariate analysis, the total dose (p = 0.0035) and the age (p = 0.0445) were found to be significant prognostic factors whereas pre-treatment, dose of cortisone, change of the dose of cortisone during therapy, latency between surgery and radiotherapy, and Karnofsky performance status were not. The results can also be illustrated in terms of median survival: the median survival of older patients amounted to 0.88 years (n = 23; > = 59 years) whereas the survival of younger patients was 1.07 years (n = 23, < 59 years). Patients having been treated completely showed a median survival of 1.05 years (n = 41) whereas patients with an incomplete treatment only survived 0.81 years in median (differences not significant using Mantel-Haensel-test).

The planned total dose of 60 Gy could be applied to 41 (89%) patients. In the remaining five, therapy had to be terminated prematurely after reaching doses of 38 Gy (1 pat.), 40 Gy (1 pat.), 56 Gy (2 pats.) and 58 Gy (1 pat.) due to a deterioration of general health status.

The dose of corticosteroids (all patients were applied dexamethasone) needed by the patients was evaluated after radiochemotherapy and compared to the doses taken before. The dexamethasone dosage was chosen regularly according to the patient's complaints and the general health status. Due to an often very fast deterioration of the patients' status CT- and MRI examinations were not performed always. Thus, we cannot exclude that in some patients a local tumor progression may have lead to termination of therapy. In 63% of the patients corticoid intake could be reduced by at least one step (compare Table 1), in a further 21% the dose remained constant, and in the remaining 16% the dose had to be increased.

Chemotherapy could be applied completely in 26 (56.5%) patients. Two of these patients had a short (< 7 days) interruption of medication, one due to a subcutaneous liquor cushion which had to be treated surgically, the other due to an episode of seizures. The mean duration of continuous temozolomide intake was 35 (6-49) days. On the other hand, chemotherapy could not be given completely in 20 patients (43%), due to a deterioration of blood cell count, worsening of general health status and liver damage. One patient died of pneumonia during a phase of bone marrow aplasia having received a radiotherapy dose of 38 Gy.

**Figure 2:**
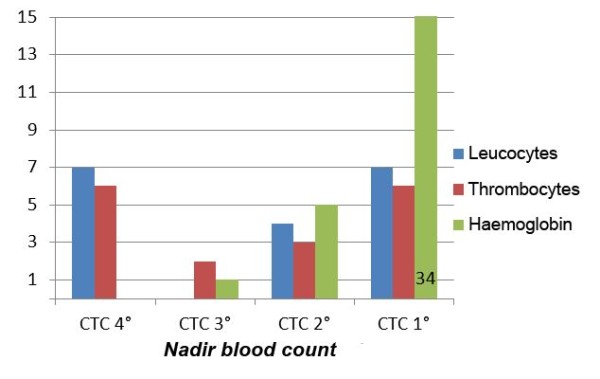
**CTC-Classification of hematologic toxicity (maximum)**.

**Figure 3:**
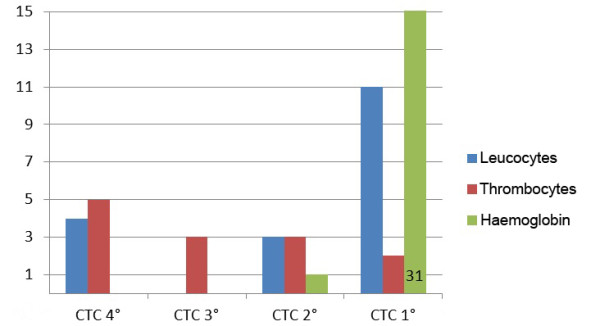
**CTC-Classification of hematologic toxicity (end of therapy)**.

The blood cell counts before and after radiochemotherapy as well as the minimal values during radiotherapy are summarized in Table 2. As stated above, the side effects were classified according to the CTC 3.0 system, resulting in the distributions depicted in Figures 2 and 3. In summary, toxicity grade three CTC to platelet count was diagnosed in two patients (4%) and grade 4 in six patients (13%), in total, six males and 2 females, whereas toxicity grade 4 CTC to white blood cell count was found in seven patients (15%, four males and three females) as the most unfavourable values during therapy. However, at the end of radiotherapy there were still three patients (7%) with grade 3 and five patients (11%) with grade 4 toxicity to the platelet count whereas only 4 patients (9%) were diagnosed to have toxicity to the leucocyte count grade 4 CTC. The duration of these side effects is summarized in Table 3.

**Table 2:** Mean blood values

	Before therapy	Nadir (minimal values during therapy)	After therapy
Hemoglobine [g/dl]	13.3 [10.4-16.7]	12.0 [7.1-15.3]	12.9 [7.1-15.3]
Leucocytes [1000/μl]	10.6 [2.2-20.5]	5.3 [0.1-11.0]	6.2 [0.2-14]
Thrombocytes [1000/μl]	227 [103-390]	141 [4-291]	166 [14-419]

**Table 3:** Duration of side effects (days)

	3° CTC	4° CTC
Thrombocytes (n = 6)	64 [26-125]	24 [14-38]

Leucocytes (n = 7)	20 [5-28]	13 [2-22]

Hemoglobine (n = 1)	4	0

Nine patients (20%) were applied erythrocyte transfusions due to symptomatic anemia (2-6 units), in six of those patients further thrombocyte transfusions were given (2-10 units). Seven patients received Filgastrim for leucopenia for on average 10 days [2-20 days].

Frequency and grade of toxicity to the liver are summarized in Table 4. In one patient, GOT, GPT and γ-GT values were elevated extremely up to values of 3172 U/l, 9020 U/l and 782 U/l, respectively. These values recovered at least in part after the end of chemotherapy. This patient was in the recovery phase of a hepatitis B infection, which was not known at the beginning of therapy. Chemotherapy was terminated due to liver toxicity in two patients.

**Table 4:** Liver toxicity at the beginning and end of therapy and maximum values

	3° CTC			4°CTC		
	**Start**	**Maximum**	**End**	**Start**	**Maximum**	**End**

γ-GT	1	3	4	0	0	0

GOT	0	1	0	0	1	0

GPT	0	4	3	0	1	0

Two (4%) of the patients had adjuvant chemotherapy using temozolomide after radiochemotherapy. This results from the fact that adjuvant chemotherapy was not applied routinely in the department at that time the patients were treated (partially before Stupp had published his trial [1]). Three patients (7%) underwent further surgery due to local relapse.

## Discussion

In the treatment of glioblastoma the effect of the combination of standard radiotherapy with simultaneous chemotherapy using temozolomide has been examined for at least 15 years. The first phase II trials were published in 1993 and 1996 [7,8]. Brock et al. [9] recommended a dosage of 75 mg/m^2^/day which is still regarded to be the standard. Very favourable survival data after radiochemotherapy were found by Stupp et al. in 2002 [10] (2-year-survival rate 31%). In the randomized trial published by the same author group in 2005 [1] the results of radiotherapy alone applying a total dose of 60 Gy in 30 fractions within six weeks were compared with the same radiotherapy plus a chemotherapy of 75 mg/m^2^/day temozolomide given simultaneously. The latter patients also received adjuvant chemotherapy with six courses of temozolomide (150-200 mg/m^2^). The combination therapy clearly improved survival (median survival 14.6 vs. 12.1 months, one-year survival rate 61.1% vs. 50.6%); since that time radiochemotherapy is regarded to be the gold standard in the treatment of glioblastoma. A second randomized trial with fewer patients was published by Athanassiou in 2005 [11]. The authors stated a median survival time of 8.9 months (radiotherapy) compared to 13.6 months (radiochemotherapy) resulting in a one-year survival probability of 15.7% vs. 56.3%.

In our collective, radiochemotherapy was terminated prematurely in 43% of the patients. This value seems high compared to those reported in the two randomized trials mentioned above: Stupp et al. [1] cite an early discontinuation of Temozolomide application in 13% of the patients, the main reason was side effects. Hematotoxicity grade 3 and 4 were noticed in 7% of the patients, mainly neutropenia and thrombopenia.

Athanassiou et al. [11] found a myelosuppression in 8.7% of their patients. Leucopenia grade 3 and 4 were noticed in 3.5% and thrombopenia grade 3 and 4 in 5.2%. One patient with severe myelotoxicity died of sepsis. Unfortunately, the authors did not state in how many patients treatment could be applied completely or how long the duration of side effects was.

Furthermore, we have found several papers showing retrospective toxicity data in the literature, see Table 5. In summary, the authors stated a rate of leucocyte toxicity grades 3 and 4 CTC in the range 3% to 15% and a frequency of thombocyte toxicity grades 3 and 4 CTC in the range 0 - 15% of their patients [12-20]. These results have been analyzed in detail, the known prognostic factors (elderly patients, unfavourable Karnofsky performance index, the percentage of patients having been operated on) did not influence markedly the rate of side effects.

**Table 5:** Literature data

Authors	Number of patients	Toxicity 3/4° leucocytes	Toxicity 3/4° thrombocytes	Remarks I	Remarks II
**Randomized trials**					

Athanassiou et al. 2005 [11]	130	3.5%	5.2%	1 pat. died of sepsis	

Stupp et al., 2005 [1]	573	4%	3%	Severe infections in 3%	

					

**Retrospective analyses**					

Armstrong 2008 [12]	203	Clinically significant lymphopenia in 45% of women and 6% of men		Abstract	

Combs et al. 2005 [26]	53	2%	0	No severe late effects	temozolomide 50 mg/m^2^

Combs et al. 2008 [14]	160	Hematologic toxicity 5% (50 mg/m^2^) vs. 14% (75 mg/m^2^)		Premature discontinuation 6.5% vs. 14%	Comparison temozolomide 50 vs. 75 mg/m^2^

Fiorica et al. 2010 [15]	42	0	5%		

Gerber 2007 [16]	52	Neutro-penia In 18%	15%	Mean duration 332 days [1-389 days]	10% required platelet transfusions, 67% discontinued radiochemo-therapy

Gerstein et al. 2009 [17]	51	Hematologic toxicity 14%		Premature discontinuation in 41%	

Jeon 2009 [27]	79	Hematologic toxicity 7.5%		1 pat. with severe lung infection	

Kimple 2010 [19]	32	3%	0		

Minniti et al. 2008 [20]	32	3%	3%	Pneumonia in 3%	

Our data	46	15%	17%		43% discontinued treatment

Some interesting case reports were also found. Nagane et al. [3], Jalali et al. [2] and Singhal et al. [4] report a total of 4 cases with long-lasting myelosuppression after temozolomide (one patient has died of sepsis).

Liver toxicity is currently under debate. While Newlands et al. [21] could not find a liver metabolism of temozolomide, liver affections were reported in literature [5,22,23]. In one case, temozolomide was applied together with valproic acid, in the two other cases a viral hepatitis was reactivated. We have seen two patients with liver toxicity which led to interruption of chemotherapy, reactivation of hepatitis could be proven in one.

Due to the low number of events prognostic factors could not be computed in our collective. Data taken from the literature show that female patients may have a higher risk of hematologic toxicity than males [24]. Armstrong et al. [25] state after having analyzed an ample collective of 680 patients that in males a body surface > 2 m^2^, missing medication with steroids, and bowel medication may enhance the risk of high-grade toxicity, while in females a creatinine level > 1 mg/dl, a platelet count below 270000/mm^3^, missing medication of gastroesophageal reflux disease and application of analgesics are independent prognostic factors.

Our data do not allow to give clear reasons for the discrepancies between our results and those taken from the literature. We only can assume that one reason may be the retrospective evaluation of an unselected patient collective performed here with a wide variety of ages, Karnofsky performance statuses and tumour extensions as well as co-morbidity. Otherwise, it may be possible that under the impression of the first long-lasting haematological side effects medication was interrupted even earlier than recommended by the manufacturer which may have lead to a virtual higher proportion of unfavourable results.

To our opinion the only strategy to limit this toxicity is to strictly follow the rules given by the manufacturer and to interrupt or to terminate the medication at the time points recommended. To our experience it would not have been possible to identify those patients with an unfavourable result before radiotherapy, thus alteration of the fractionation protocol, reduction of the dose of temozolomide prematurely or even not to apply temozolomide concomitantly to radiotherapy do not appear to be reasonable possibilities.

## Conclusions

Our values fit well to those taken from the retrospective analyses. However, the results in the randomized trials are far more favourable which may be a result of patient selection. Unfortunately, the known prognostic factors could not be tested in our collective.

Bearing in mind the limited power of a retrospective evaluation we would like to conclude that simultaneous radiochemotherapy for glioblastoma yields reasonable local control and survival rates. However, a certain risk of toxicity to the bone marrow must be taken into account.

According to the recommendations given by the manufacturer should be interrupted if neutrophile count is between 0.5 - 1.5 × 10^9^/l or thrombocyte count is between 10 and 100 × 10^9^/l or non-hematologic toxicity reaches CTC grade 2. Below these blood cell counts or in case of non-hematologic toxicity CTC grades 3 and 4 medication should be terminated.

## List of abbreviations

CTC: Common toxicity criteria [6].

## Competing interests

The authors declare that they have no competing interests.

## Authors' contributions

MN supervised data acquisition and evaluation and wrote the manuscript. CB collected the data and performed a part of the evaluation. This paper is a short version of Dr. CB's thesis. JF was responsible for radiotherapy of the patients and reviewed the manuscript. NL was responsible for the physical part of radiotherapy.

RK was responsible for surgery of the patients and reviewed the manuscript. CR supervised radiotherapy of the patients and reviewed the manuscript. All authors have read and approved the final manuscript.
